# How healthcare systems are experienced by autistic adults in the United Kingdom: A meta-ethnography

**DOI:** 10.1177/13623613241235531

**Published:** 2024-03-11

**Authors:** Sarah Radev, Megan Freeth, Andrew R Thompson

**Affiliations:** 1 Cardiff University, UK; 2 The University of Sheffield, UK; 3 Cardiff and Vale University Health Board, UK

**Keywords:** adults, autism spectrum disorders, autistic, health services, healthcare, qualitative research, UK healthcare

## Abstract

Autistic adults are at increased risk of both mental and physical health difficulties, and yet can face barriers to accessing healthcare. A meta-ethnographic approach was used to conduct a review of the existing literature regarding autistic adults’ experiences of accessing healthcare. Four databases were systematically searched for qualitative and mixed-method studies reporting on the experiences of autistic adults without a co-occurring learning disability accessing adult healthcare services within the United Kingdom. Fifteen studies met the inclusion criteria, and seven steps were used to systematically extract the data and then generate novel themes. Three superordinate themes were identified: *Professionals’ lack of knowledge can be damaging*, *Need to reduce processing demands* and *Adaptation to improve engagement.* This review highlights the wide-reaching damaging impact misdiagnosis, inadequate or inappropriate treatment, overwhelming environments and inaccessible systems can have on the well-being and ability of autistic adults to engage with treatment. The lack of autism knowledge and understanding experienced in interactions with healthcare professionals, along with autistic adult’s own communication and sensory processing differences, demonstrates the need for widely delivered training co-produced with autistic adults alongside bespoke and person-centred adaptations.

**Lay abstract:**

Autistic adults are more likely to experience mental and physical health difficulties, and yet can find it difficult to get the support that they need. A meta-ethnographic approach was used to review the existing research on autistic adults’ experiences of accessing healthcare. Four databases were searched for qualitative and mixed-method studies which looked at the experiences of autistic adults who did not also have a learning disability when using healthcare services in the United Kingdom. Fifteen papers met the criteria to be included, and seven steps were used to analyse the information and develop new themes. Three main themes were identified: *Professionals’ lack of knowledge can be damaging*, *Need to reduce processing demands* and *Adaptation to improve engagement.* This review highlights how damaging misdiagnosis, inappropriate treatment, overwhelming environments and systems that are difficult to access can have on the well-being of autistic adults. Limited knowledge and understanding about autism knowledge among healthcare professionals along with autistic adult’s own communication and sensory differences indicate that there is a need for improved training developed with autistic adults and adaptations.

## Introduction

Increased levels of both physical and mental health difficulties have been identified in the autistic population compared to the non-autistic population, leading to an overall poorer quality of life (Buck et al., 2014; Caamaño et al., 2013; Gilmore et al., 2022). Reduced satisfaction with health services and higher levels of unmet needs have been reported by autistic adults when compared to the needs of non-autistic adults (Brede et al., 2022; Raymaker et al., 2017).

A number of studies have investigated autistic adults’ experiences of healthcare, some of which have focused on mental health settings (Brede et al., 2022) and some of which have focused on physical health settings (Calleja et al., 2020; Mason et al., 2019; Walsh et al., 2020). The key themes that have most commonly arisen include difficulties with communication (Brede et al., 2022; Nicolaidis et al., 2015; Raymaker et al., 2017; Shaw et al., 2023); sensory sensitivities (Brede et al., 2022; Brice et al., 2021; Nicolaidis et al., 2015; Raymaker et al., 2017; Shaw et al., 2023); a lack of knowledge about autism (Brede et al., 2022; Brice et al., 2021; Jones et al., 2014; Nicolaidis et al., 2015) and difficulties with accessing services (Brede et al., 2022; Nicolaidis et al., 2015; Raymaker et al., 2017; Shaw et al., 2023). Other themes that have come up to a lesser extent include difficulties with slower processing speeds (Nicolaidis et al., 2015; Raymaker et al., 2017); feelings of helplessness and self-doubt (Shaw et al., 2023); emotional regulation (Brede et al., 2022; Raymaker et al., 2017); and difficulties with the need for flexibility and certainty (Brede et al., 2022; Shaw et al., 2023) all of which are known difficulties faced by autistic people. Some papers have focused solely on the autistic experience, while others have included perspectives from professionals and carers. Considering that one of the key recurring themes highlighted by autistic people is a feeling that professionals have a lack of knowledge about autism, the inclusion of the professionals’ views may not be representative of the experiences of autistic adults. For this reason, this meta-ethnography will maintain a focus on the direct experiences of autistic adults from their own perspectives.

With the exception of Gilmore et al. (2022), which focused on experiences of the US healthcare system, previous systematic reviews have included papers from a mix of different countries including Europe, the United Kingdom, the United States and Canada where the UK data is considered alongside wider global data, rather than being UK specific. This is important since the healthcare systems in these different countries are very different not only in the ways they are set up, but in how they are accessed by the population. To date, there have been no systematic reviews that have specifically focused on the experiences of autistic adults accessing healthcare services in the United Kingdom alone.

The current study approaches this issue broadly from a critical realist perspective, and the analysis is deliberately interpretivist, in keeping with the meta-ethnographic approach and the aim to go beyond simply summary of the extant literature. Meta-ethnography is the most widely used synthesis methodology for qualitative studies on health and social care (France et al., 2019) and provides an opportunity to summarise extant qualitative studies while enabling new findings, which in this case may assist in gaining a greater understanding of the experience and needs of autistic people seeking services in the United Kingdom.

## Method

The protocol of this review was developed in line with the most recent PRISMA (Preferred Reporting Items for Systematic Reviews and Meta-Analyses) guidelines (Page et al., 2021) and was registered on PROSPERO (International Prospective Register of Systematic Reviews – Registration #:CRD42022332477).

### Search strategy

The search strategy was developed in consultation with a librarian subject expert. Search terms were combined with Boolean operators: (‘interpretative phenomenological analysis’ OR ‘grounded theory’ OR ‘constructivist epistemological framework’ OR ‘semi structured’ OR unstructured OR informal OR indepth OR ‘face to face’ OR structured OR guide* OR interview* OR discussion* OR questionnaire* OR ‘focus group*’ OR qualitative or ethnograph* OR ‘field work’ OR fieldwork OR ‘key informant’) AND (autis* OR asd OR asc OR aspie OR asperger*) AND (Health Services OR health* OR practice) AND (experience* OR perspective* OR attitude* OR view* OR opinion*). Four databases MedLine, PsychInfo, Scopus and PubMed were searched initially in December 2022 and again on 10 January 2024.

### Eligibility criteria

Following the removal of duplicate papers, the Rayyan software (Ouzzani et al., 2016) was used to conduct automated searches to eliminate studies based outside the United Kingdom, and which were not in the English Language. Titles and abstracts were then reviewed in line with inclusion and exclusion criteria which specified studies should have collected qualitative data from autistic adults without a learning disability about accessing healthcare services in the United Kingdom, before full texts were examined. Studies which included adults with a learning disability were excluded to ensure that experiences of healthcare related to being autistic, rather than to having a learning disability. Studies where some participants were based outside the United Kingdom, where there was a combination of reports from autistic adults, family members and professionals or which focused on the transitional period from child to adult services were excluded. Where it was possible to extract the data of autistic adults from the results, where reports of other parties such as professionals or autistic adults accessing healthcare in other countries were included, the paper was not excluded, such as Mason et al. (2021) and Bruce et al. (2023).

**Figure 1.**
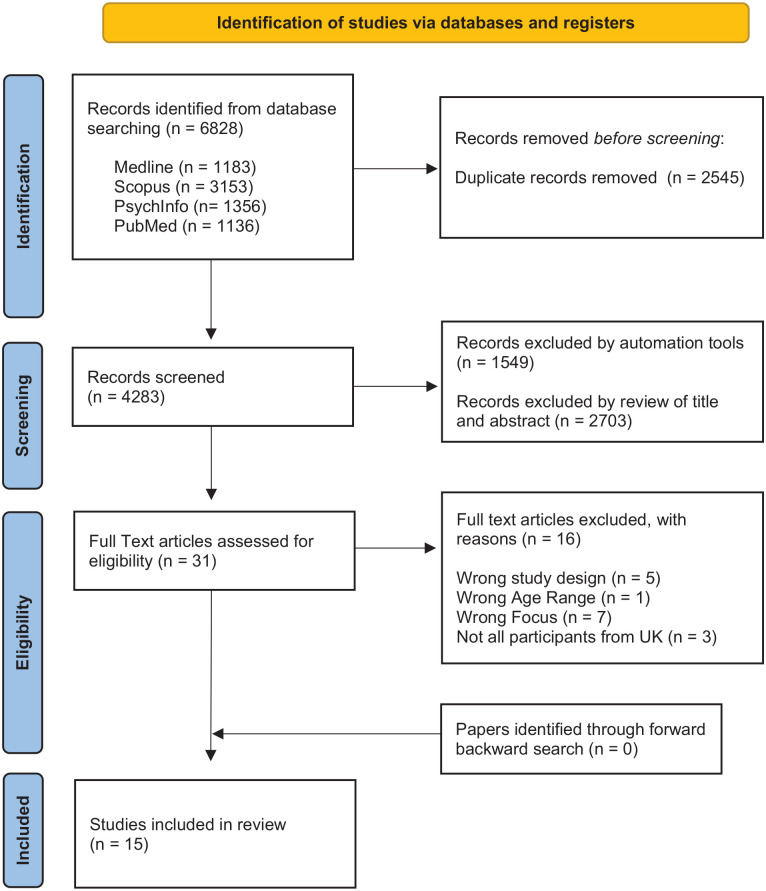
PRISMA flow diagram for systematic literature search of autistic adults’ experiences of healthcare in the United Kingdom.

Reference lists of all included papers were manually searched, but no further relevant papers were found. An outline of all included papers can be found in Table 1. The full process of selection can be seen in the PRISMA diagram in Figure 1 (Page et al., 2021) and involved discussions with all authors at each step.

**Table 1.** Summary of the main characteristics of all studies included for synthesis.

Study	Authors	Quality appraisal CASP score	Focus	Participant characteristics	Method of data collection	Method of analysis	Key themes	Approach
1	(Ali et al., 2023)	7.5	Telehealth experiences of UK-based autistic adults following the COVID-19 pandemic	*N* = 11 autistic adults aged 26–67 *N* = 7 family members or carers *N* = 6 healthcare professionals	In-depth interviews	Thematic analysis	1. Technology aids communication and access – except when it doesn’t 2. Inflexibility	Interpretive approach where researchers viewed the data through the lens of their own biopsychosocial experience
2	(Au-Yeung et al., 2019)	8	Autistic adults’ reports of receiving mental health diagnosis and the degree to which they agree with them	Three participant groups *N* = 208 autistic *N* = 71 non-autistic and possibly autistic	Survey with both closed- and open-ended questions	Thematic analysis	1. ‘Problems with Mental Health Diagnosis’ 2. ‘Clinical Barriers’	Participatory interpretive approach
3	(Babb et al., 2021)	8.5	Autistic womens’ experience of accessing eating disorder services	Three participant groups *N* = 15 autistic women with experience of AN, parents and healthcare professionals	Interviews	Thematic Analysis	1. Misunderstanding autism and autistic traits 2. One treatment does not fit all 3. Improving accessibility and engagement within services	Participatory interpretive approach
4	(Bruce et al., 2023)	9	Exploring the Experiences of Autistic Transgender and Non-Binary Adults in Seeking Gender Identity Health Care	Transgender autistic adults aged 18–46 *N* = 17 (3 from UK)	Interviews	Thematic Analysis	1. Perceived lack of professional knowledge 2. Accessibility issues 3. Bureaucracy and Economic barriers	Participatory interpretive approach
5	(Camm-Crosbie et al., 2019)	9	Autistic adults’ experiences of treatment and support for mental health problems, self-injury and suicidality	Autistic adults registered with the Cambridge Autism Research Database. *N* = 122 females *N* = 77 male *N* = 1 unreported	Online survey with open-ended questions	Thematic analysis	1. ‘People like me don’t get support’ 2. Lack of understanding and knowledge 3. Well-being	Exploratory qualitative approach from an essentialist theoretical position
6	(Crane et al., 2018)	7.5	Autism diagnostic experiences in the United Kingdom	10 diagnosed autistic adults aged 29–59 years old (42.89 mean age.) *N* = 6 females *N* = 4 males	Semi-structured interviews	Thematic analysis	1. The process of understanding and accepting autism 2. Barriers to satisfaction with the diagnostic process 3. Inadequate post-diagnostic support provision	Essentialist framework using an inductive approach
7	(Griffith et al., 2012)	7.5	Experiences of support among individuals in middle adulthood with Asperger syndrome	11 diagnosed autistic adults over 35 years old (46.45 mean age). *N* = 7 males *N* = 4 females	Semi-structured interviews	IPA	1. ‘Some days I do struggle’ – living with Aspergers 2. They don’t expect you to have problems with things – employment issues 3. ‘I just fall through the gaps between’ – experiences with mainstream support 4. ‘Raising awareness’ – Future steps towards supporting people with Asperger syndrome	Essentialist framework using an inductive approach
8	(Harmens et al., 2022)	8.5	The relationships between diagnosis, well-being and identity in autistic women	*N* = 24 autistic women at various stages of diagnosis	Semi-structured interviews following on from wider survey	Thematic analysis	1. Don’t forget I’m autistic 2. What now? 3. Having to be the professional 4. No one saw me.	Interpretive approach using inductive reflexive analysis
9	(Leedham et al., 2020)	9	Experiences of autistic females who receive a diagnosis in middle to late adulthood	*N* = 11 women diagnosed autistic at or after the age of 40	Interviews	Thematic analysis	1. A hidden condition 2. The process of acceptance 3. Post diagnostic impact of others 4. A new identity on the autism spectrum	Interpretive approach to understanding participant experiences
10	(Mason et al., 2021)	7.5	To explore healthcare experiences from the perspective of both autistic people and clinicians	Self-reported diagnosed autistic adults age range 29–65 years old *N* = 6 males *N* = 5 females	3 × focus groups	Framework analysis	1. Cognitive factors 2. Patient characteristics 3. Healthcare professionals perceived knowledge 4. Healthcare provision 5. Adjustments to healthcare 6. Autism diagnosis	Interpretive inductive approach
11	McMillion et al., 2021)	7	Dental experiences of UK-based autistic adults	Autistic (*n* = 37) and non-autistic adults accessing the dentists *N* = 27 female *N* = 9 male *N* = 1 other	Survey including open-ended questions.	Thematic analysis	1. Interaction with dental practitioners 2. Preparedness 3. Challenging Sensory environment 4. Anxiety 5. Pain	Interpretive inductive approach to analyse responses at a semantic and informative level
12	(Parmar et al., 2022)	7.5	Exploring barriers and facilitators to accessing optometry services for autistic adults without learning disabilities	Autistic adults aged 25–67 (mean age 47.1) *N* = 6 female *N* = 12 male	Focus group	Thematic analysis	1. Practice operation 2. Eye examination-specific considerations 3. Patient-practitioner relationship 4. Preparing the patient for their visit	Interpretive inductive category formation approach
13	(Pentz et al., 2023)	8	Experiences of women with autistic spectrum condition accessing the Brighton and Hove Specialist Perinatal Mental Health Service	Autistic adults who were patients of the Brighton and Hove Specialist Perinatal Mental Health Service. *N* = 5	Semi-structured interviews	Thematic analysis	1. Interventions 2. Support of ASC characteristics 3. Practitioners Support	Interpretive narrative approach
14	(Punshon et al., 2009)	8	Experiences of adults with Asperger syndrome relating to their diagnosis; and how services might help individuals negotiate the diagnostic process	*N* = 10 adults with Aspergers	Semi-structured interviews	IPA	1. Negative life experiences 2. Experiences of services (pre-diagnosis) 3. Beliefs about symptoms of Asperger syndrome 4. Identity formation 5. Effects of diagnosis on beliefs 6. Effect of societal views of Asperger syndrome	Interpretive phenomenological approach influenced by phenomenology and poststructuralist thought
15	(Talcer et al., 2023)	9	To explore the sensory experiences of autistic mothers and their impact on motherhood	*N* = 7 autistic women	Interviews	Thematic analysis	1. Antenatal 2. Sensory experiences in motherhood 3. Impact 4. Strategies and needs 5. Diagnosis	Interpretive inductive approach

### Quality appraisal process

The methodological quality of the included papers was assessed using the Critical Appraisals Skills Programme (CASP Qualitative Checklist, 2018) checklist. Studies were graded ‘low’ (8.5 or higher), ‘medium’ (5–8) or ‘high’ (less than 5) in relation to the risk of having significant methodological flaws. No studies were excluded based on their rating, and no papers received a rating indicative of having a high likelihood of significant methodological flaws. Once initial full-text articles had been agreed by the authors of this article, an independent researcher who was not part of the research team separately evaluated 50% of the full-text articles with an initial 62% agreement. Following discussions between the first author and the independent researcher regarding those papers on which agreement was not initially reached 92% agreement was achieved.

### Data synthesis and theme generation

The meta-ethnography seven-phase method developed by Noblit and Hare (1988) was used for synthesising data and generating new themes. This process was informed by Britten et al.’s (2002) use of first-, second- and third-order constructs where the first order represents opinions of the original participants, the second order represents the interpretations of the authors of the different studies and the third order represents new interpretations of the second-order constructs by this author (Schutz, 1962).

### Community involvement

The first author is herself an autistic adult living in the United Kingdom and accessing the UK healthcare system.

## Results

### Summary of themes

This meta-ethnography identified three superordinate themes relating to the experiences of autistic adults in the United Kingdom when accessing healthcare: Professionals’ lack of knowledge can be damaging; need to reduce processing demands; and adaptations to improve engagement. Each superordinate theme contained further subthemes which are represented in Table 2.

**Table 2.** Summary of third- and second-order constructs.

Super-ordinate third-order construct	Sub-ordinate third-order construct	Second-order construct(s)
Professionals’ lack of knowledge can be damaging	Misunderstanding leading to misdiagnosis and inadequate support	• Autism not recognised • Misdiagnosed with other conditions • Spectrum nature of autism causes misunderstanding of needs • Failed interventions
	Pressure to be the expert in the room	• Tension on whether the individual or the professional is ‘the expert’ • Lack of professional guidance can lead to self-doubt
Need to reduce processing demands	Communication differences	• Alexithymia makes explaining symptoms difficult • How information is presented is important • Too much verbal communication can be overwhelming • Anxiety negatively impacts ability to communicate
	Sensory processing differences	• Clinical environments should be appropriate • Sensory reactivity can cause distress
Adaptations to improve engagement	Accessibility difficulties	• Support needs to be pitched at the right level • Difficulties accessing appointments • Diagnostic pathways are unclear
	Need for consistency and predictability	• Autistic adults need to know what to expect from an appointment • Consistency is important

### Theme 1: professionals’ lack of knowledge can be damaging

All papers reported that autistic adults’ experienced healthcare professionals as not having a good understanding of autism most of the time.

#### Misunderstanding leading to misdiagnosis and inadequate support

Participants in some studies reported their difficulties were not appropriately attributed to or recognised as autistic characteristics and were instead misunderstood as mental health symptoms (Bruce et al., 2023; Griffith et al., 2012). An example of this misattribution of autism characteristics was found in eating disorder services, where they were seen as being driven by the eating disorder, rather than being understood as sensory sensitivities:

When I was in hospital, I kept getting told off for walking on tip toes and for fidgeting a lot . . . they thought I was doing these things to burn more calories, except I’d been doing them for as long as I could remember. (Babb et al., 2021)

Similarly, communication difficulties were interpreted as a reluctance to engage in treatment and in some cases as ‘rudeness’. In more severe cases, a lack of eye contact was reported as being understood as an indication of psychosis rather than the social communication style (Au-Yeung et al., 2019). Several studies discussed this issue having arisen in the context of late diagnosis:

I had a formal diagnosis of bipolar (II) condition for around 17 years. I actually satisfy the DSM criteria for this, but have only [ever] been hypomanic once and that was antidepressant induced . . . once my autism had been diagnosed, I was able to get a consultant psychiatrist to say that she didn’t think I’d ever had bipolar and it has been revoked as a current diagnosis. (Au-Yeung et al., 2019)

Such delays in receiving the correct diagnosis were reported as contributing to autistic adults’ sense of blame and feeling misunderstood (Leedham et al., 2020; Punshon et al., 2009). Another issue evident in the extant literature and linked to poor understanding was a sense of professionals underestimating the level of need, as one participant commented, ‘I went to my GP, and . . . he started off saying, because I go to work every day, that means everything’s fine . . .’ (Harmens et al., 2022). In other cases, it was felt that a lack of understanding resulted in their autistic needs being overlooked as ‘there was a lack of understanding or experience and they didn’t want to sort of try and open up something that maybe they didn’t know much about’ (Pentz et al., 2023) which ultimately resulted in autism-specific support needs being overlooked.

Participants also reported encountering outdated and stereotyped understandings of autism which again impacted on the professionals’ understanding and perception of what support they needed and in some cases served as a barrier to autism diagnosis (Babb et al., 2021; Crane et al., 2018; Griffiths et al., 2019; Mason et al., 2021).

The lack of understanding of the spectrum nature of the condition among healthcare professionals is reported in the literature reviewed as either being viewed as not being autistic or being labelled as ‘high functioning’ and therefore requiring less or no support (Camm-Crosbie et al., 2019):

I’m on the high-functioning end [of the autistic spectrum] and so I don’t fit mental health, I don’t fit learning disability. I just fall through the gaps between departments, whether it’s in the health service or social services. (Griffith et al., 2012)

Such misconceptions and misattribution resulting from a lack of knowledge about autism can be damaging, not only in the impact on well-being and sense of self, but in the treatment that is then prescribed and/or provided. In some cases, misdiagnosis was reported as having led to inappropriate use of medication:

I was given anti-psychotics for my behaviour . . . they think because [I don’t] look at them and [I] am nervous of talking then [I] am ‘guarded’ or have ‘flat affect’. (Au-Yeung et al., 2019)

#### Pressure to be the expert in the room

Several studies contained accounts of participants feeling they had to bridge the gaps in healthcare professionals’ knowledge and be the experts in the room, needing to ‘teach’ professionals how to support them (Talcer et al., 2023). This pressure to be the expert presents a nuanced tension that requires careful negotiation between the healthcare professional and the autistic person:

it feels very much like the onus is on you as an individual to make the assessment happen, rather than a medical practitioner saying ‘this is something that we should see’. (Harmens et al., 2022)

This patient-professional tension regarding who is the expert in the room was also referenced as causing autistic adults to start to doubt themselves and their own understanding of their differences due to the imbalance of power:

. . . got to a point where I was almost convinced that they wanted to be right and I’d actually buried some deep trauma and I had no memory of it and I started questioning the whole – like everything. (Leedham et al., 2020)

This power imbalance and the harm that could be caused due to professional’s lack of autism knowledge and understanding can place a great deal of stress and pressure on the autistic individual.

### Theme 2: need to reduce processing demands

It was evident in the studies reviewed that many participants had experienced services as placing too many processing demands upon them, both in the form of communicative information and sensory information presented.

#### Communication differences

Many of the studies identified communication differences as a key theme. Participants talked about difficulties with the way professionals interacted with them (Au-Yeung et al., 2019), which impacted on their ability to process and understand information. Participants advised they prefer it if professionals ‘don’t ask rapid fire questions’ (McMillion et al., 2021) and that ‘if they speak quickly, they might as well not bother’ (Parmar et al., 2022).

Participants reported particularly struggling with answering subjective or vague questions due to difficulties with tuning into the subtleties of feelings in their body (Mason et al., 2021) and with alexithymia (Camm-Crosbie et al., 2019; Punshon et al., 2009):

opticians don’t tell you that. They just say ‘can you see a difference’ but they don’t tell you that you are supposed to be aiming for a point when there is no difference . . . it is very stressful for the autistic person because you are looking for a difference that isn’t there. (Parmar et al., 2022)

Studies reported on the negative impact this had resulting in many feeling ‘mentally tired with all the questions’ and feeling as though they were getting it wrong and ‘giving them the wrong answer’ and thereby making things worse (Parmar et al., 2022).

In several studies, participants expressed a need for the language used to be concrete and explicit so that they knew what to expect and to prevent misunderstandings due to literal interpretations (Mason et al., 2021; Parmar et al., 2022; Punshon et al., 2009):

You want to tell them things, but your mind empties, you can’t find the right words . . . I know he [the clinician] doesn’t want the literal answer . . . ‘What brings you here today?’, I know he doesn’t want to hear ‘the bus’. (Mason et al., 2021)

Many of the studies also discussed the use of alternative methods of communication to aid understanding such as visual aids, written information or video guides. For example, Parmar et al. (2022) reported that some of their participants expressed preferring email communication because it meant that they had communication trails they could utilise.

In addition to these communication differences, autistic adults talked about the impact anxiety could have on the ability to communicate effectively. This may be due to building anticipation of the appointment beforehand or could be triggered by sensory sensitivities in the environment, or feeling rushed in the appointment (McMillion et al., 2021):

Well, anything that overloads my head, my brain just shuts down then . . . I have to take my wife everywhere and she has to tell them to go away. (Parmar et al., 2022)

If too many questions are asked, or if questions are asked that are unexpected and which the individual does not have ready pre-prepared answers for, it can be too much to process and can become overwhelming (Mason et al., 2021). One participant explained that ‘if unexpected questions arise, it throws my head in a tailspin’ (McMillion et al., 2021).

#### Sensory processing differences

Negative experiences of overwhelming environments were reported to contribute to patient dissatisfaction with healthcare services (Babb et al., 2021; Crane et al., 2018; McMillion et al., 2021). For many participants, this experience of sensory discomfort began from the waiting area which was often experienced as bright, noisy and busy (Mason et al., 2021; McMillion et al., 2021). One participant shared that ‘the light, my goodness, it was too much’ (Crane et al., 2018) while another explained that ‘bright lights cause migraines’ (McMillion et al., 2021). Often these statements are made in relation to the bright lights of the hospital environment, but participants also referred to the difficulties that bright lights created during medical assessments:

I didn’t like the ones where you’ve got bright lights in your eyes like the flash and this machine here [slit lamp]. I sort of had to tense myself to cope with it . . . it was at the edges of what I could tolerate. (Parmar et al., 2022)

Participants also talked about negative experiences with noise in medical settings which can be ‘intense’ and ‘upsetting’ and how it is ‘hard to block them out even with headphones or ear plugs’ (McMillion et al., 2021). Often it was not a single loud noise that was problematic, but a multitude of noises.

Touch was also a particular issue for many participants, especially when examinations involved the clinician needing to get physically close. Participants expressed it is ‘beyond uncomfortable’ (Parmar et al., 2022) and ‘unpleasant being touched by a stranger’ (McMillion et al., 2021):

It (labour) was difficult especially when you are having to be touched by medical people, I don’t like being touched at all by anybody . . . It’s hard to explain but it hurt . . . It was horrific. (Talcer et al., 2023)

The proximity of medical equipment was also identified as being problematic and ‘quite intrusive’ (Parmar et al., 2022). Participants also experienced difficulties with the smell of environments including not only medical smells but also strong perfumes worn by practitioners (Parmar et al., 2022).

### Theme 3: adaptations to improve engagement

The third superordinate theme ‘adaptations to improve engagement’ looks at how autistic adults’ experiences of accessing healthcare can inform adaptations to services to increase accessibility and engagement.

#### Accessibility difficulties

Difficulties with accessing services was a theme that came up in several studies beginning as early in the process as booking appointments. Many shared this was particularly difficult to do if a phone call was required preferring ‘other ways to make appointments than phoning’ (McMillion et al., 2021) due to feeling ‘really uncomfortable on the phone’ (Parmar et al., 2022). Communicating on the phone was highlighted as particularly problematic for some because ‘socially, I can use some visual cues, and the phone takes that away’ (Ali et al., 2023). One participant recounted how despite acknowledging they were waiting for an autism assessment ‘did not ask if there was anything they could do to make the phone call easier’ (Bruce et al., 2023). These difficulties with talking to healthcare staff on the phone also had a detrimental impact on the individuals’ well-being due to the associated anxiety (Harmens et al., 2022). For some, there was a preference to book appointments in person rather than on the phone:

I write down what days I’m available, what times I’m available, and I say, I need an eye appointment, there’s the information, fit me in somewhere around that. (Parmar et al., 2022)

The stress that built up prior to attending appointments was described by many as another barrier and often resulted in appointments being cancelled, avoided until they were unavoidable or until a number of problems had accumulated (Mason et al., 2021). One participant commented, ‘I feel very anxious about going . . . I often cancel appointments or put off going for as long as possible’ (McMillion et al., 2021).

Online booking systems were highly praised as ‘absolutely brilliant’ and some participants found it increased their likelihood of accessing healthcare services (Ali et al., 2023) because ‘if I can book online then I don’t avoid making appointments for four months’ (Parmar et al., 2022). One study highlighted how technology can be used to aid accessibility and engagement with healthcare through the option of virtual appointments:

if I was at work and I needed an appointment, I wouldn’t have my car and then I’d have to sort out or change my routine, and, as an autistic person, it’s a huge change to my routine that I don’t like, I don’t want to deal with. It kind of removes that stress. (Ali et al., 2023)

#### Consistency and predictability

Another element discussed in the studies regarding adaptations to improve autistic adults engagement was to ensure consistency and predictability as changes could be ‘unsettling’ (Parmar et al., 2022). Many autistic adults talked about the importance of consistency or continuity of care to them (Mason et al., 2021; McMillion et al., 2021; Talcer et al., 2023), particularly in relation to consistency of the professionals they saw because it allowed them to build rapport with and trust in the professional:

I think for anyone, but especially for someone who’s autistic I think that having continuity of care is so important. (Pentz et al., 2023)

In addition to needing consistency, studies highlighted how participants favoured predictability and being informed about both the environments they would be entering and the procedures they would be involved in ahead of their appointments. For many, this was a really important factor in reducing anxiety and helping them to access services:

now I’ve got the information, I’m not anxious about it anymore . . . And it’s a tiny little adjustment from their end, but it lowers my anxiety on a day-to-day basis. (Parmar et al., 2022)

The way this information was provided was important. For some participants, the preference was to ensure that ‘everything is in writing’ (McMillion et al., 2021) while others preferred it to be presented visually in the form of pictures or videos so that they ‘didn’t have to guess what the tests or equipment may be like’ (Parmar et al., 2022):

it was just like ‘Oh, they really understand my needs’ because they had photos and biographies of everyone you were likely to meet, they had photos of all of their consulting rooms, a map, a photo of the outside of the building, so it was as if they anticipated all of the things that were likely to worry me. (Harmens et al., 2022)

These adaptations and pre-emptive planning of supporting autistic adults to be informed of their appointments in advance was experienced as extremely important for reducing anxiety and thereby supporting engagement (Babb et al., 2021; McMillion et al., 2021; Parmar et al., 2022):

I never would have thought, prior to finding out about my autism and everything, that this sort of stuff would be helpful for me . . . I’m surprised much how less anxious I feel. (Parmar et al., 2022)

## Discussion

This meta-ethnography aimed to synthesise existing data regarding autistic adult’s experiences of healthcare in the United Kingdom and look to generate new insights into this experience. Previous reviews have focused on global experiences, while the intention here is to specifically examine experience within the UK healthcare system, to be able to make recommendations for use in this context. The meta-ethnography revealed three superordinate themes: ‘professionals’ lack of knowledge can be damaging’, ‘need to reduce processing demands’ and ‘adaptations to promote engagement’.

The first theme ‘professionals’ lack of knowledge can be damaging’ contained two subthemes, each looking at a different way in which a lack of knowledge among healthcare professionals can be experienced as damaging to autistic adults. The theme of healthcare professionals lacking knowledge is one that has often been raised by autistic adults in previous research, but through this meta-ethnography what came across was the wide-reaching damage that can be done by professionals not having an adequate understanding of autism when treating autistic adults. This damage can arise through the misdiagnosis that can result from misunderstanding difficulties and the resulting pressure it places on the autistic adults themselves to be the expert in the room.

The data in these studies related to experiences of encountering a lack of autism knowledge from healthcare professionals resulting in autistic characteristics being misattributed or misunderstood as characteristics of alternative conditions, which ultimately led to the wrong diagnoses being given (Au-Yeung et al., 2019; Babb et al., 2021; Bruce et al., 2023; Griffith et al., 2012) and an autism diagnosis being delayed (Griffith et al., 2012; Leedham et al., 2020; Mason et al., 2021; Punshon et al., 2009). Participants also reported encountering misconceptions regarding the spectrum nature of the condition, which meant that being classed as ‘high functioning’ and therefore able to cope resulted in inadequate support being offered (Camm-Crosbie et al., 2019; Griffith et al., 2012; Harmens et al., 2022). When treatment or support was offered, a lack of understanding of autism also resulted in what was offered being inappropriate for autistic individuals and potentially doing more damage than good (Babb et al., 2021; Camm-Crosbie et al., 2019; Crane et al., 2018; McMillion et al., 2021; Pentz et al., 2023).

These findings are in line with previous findings that a lack of knowledge among healthcare professionals can lead to misdiagnosis, especially where autism and mental health was confused, delayed autism diagnosis and inappropriate treatment which can ultimately have a negative impact on the individuals well-being and serve as a barrier to receiving the right support (Bradshaw et al., 2019; Brede et al., 2022; Wilson et al., 2023; Yau et al., 2023). This lack of understanding among healthcare professionals highlights the importance of co-production with autistic adults of appropriate training.

Such co-production would begin to address the perceived pressure on autistic adults to be the experts in the room, to educate professionals on the way systems worked and hold the responsibility of proving themselves (Camm-Crosbie et al., 2019; Crane et al., 2018; Griffith et al., 2012; Harmens et al., 2022; Leedham et al., 2020). Participants expressed feeling it was not their place to fill in the gaps of inadequate training, and the additional stress this placed on them was damaging to both their mental health and their ability to access appropriate healthcare pathways.

The second superordinate theme ‘need to reduce processing demands’ includes the subthemes of ‘communication differences’ and ‘sensory processing differences’, which are two themes that have consistently come up in previous research. In regard to the first subtheme ‘communication differences’, participants talked about difficulties in processing information during verbal communication (Au-Yeung et al., 2019; McMillion et al., 2021; Parmar et al., 2022) which was exacerbated by feelings of anxiety (Camm-Crosbie et al., 2019; Mason et al., 2021; McMillion et al., 2021; Parmar et al., 2022). Participants reported finding it difficult to answer subjective questions and being aware that their literal interpretations could cause difficulties (Camm-Crosbie et al., 2019; Mason et al., 2021; Parmar et al., 2022; Punshon et al., 2009). The use of alternative means of communication including video, phone calls, and email was beneficial for some, but not for others, emphasising the need for a bespoke approach (Ali et al., 2023; Harmens et al., 2022; Parmar et al., 2022). These findings are in line with previous papers which have identified that autistic adults can struggle with misunderstandings when communicating, have a preference for written communication and find it difficult to express emotions and answer open questions, which can be exacerbated due to feelings of stress and being overwhelmed (Bradshaw et al., 2019; Brede et al., 2022; Mason et al., 2019; Walsh et al., 2020).

Sensory processing differences were identified in several papers as a way in which participants could become overwhelmed, as is represented in the second subtheme under ‘need to reduce processing demands’. These findings are in line with those found in previous papers where these sensory differences were noted to act as barriers to accessing healthcare, to impact on communication and to be misinterpreted as symptoms of mental health difficulties rather than as autistic characteristics (Bradshaw et al., 2019; Brede et al., 2022; Mason et al., 2019; Walsh et al., 2020). This article adds to the existing literature by considering more directly the combined impact that sensory processing differences and communication differences can have on each other as each increases levels of anxiety which can then reduce ability to cope with either. Taking into consideration the number of demands placed on autistic people to process different input throughout a healthcare visit and keeping this to a minimum may be an important way to ensure they do not become overwhelmed.

The final superordinate theme ‘adaptations to improve engagement’ examined how autistic experiences can inform adaptations to improve accessibility and engagement with services. Difficulties with accessibility came up across several studies beginning with difficulties with appointment booking systems and anxiety regarding attending appointments (Ali et al., 2023; Babb et al., 2021; Bruce et al., 2023; Harmens et al., 2022; Mason et al., 2021; McMillion et al., 2021; Parmar et al., 2022). The need for services for autistic people being ‘bespoke’ and ‘evidence based’ has been highlighted in previous articles (Brede et al., 2022) as well as the increased anxiety experienced when accessing services (Bradshaw et al., 2019).

The subtheme ‘need for consistency and predictability’ demonstrates how simple adaptations can be made to increase engagement through increasing consistency and predictability. The need for ‘restricted, repetitive patterns of behaviour, interests or activities’ is one of the diagnostic criteria for autism (American Psychiatric Association, 2013) and can result in insistence on sameness and inflexibility. Therefore, it is not surprising that participants talked about the importance of consistency of staff and environments (Camm-Crosbie et al., 2019; Mason et al., 2021; Parmar et al., 2022; Pentz et al., 2023) and of being fully informed of environments and procedures prior to appointments to increase predictability (Harmens et al., 2022; Mason et al., 2021; McMillion et al., 2021; Parmar et al., 2022). This need has not widely been reported as an overarching theme in previous papers, but Brede et al. (2022) do refer to the need for predictability.

### Clinical implications

This meta-ethnography has highlighted the wide-reaching damaging impact that a lack of autism knowledge and understanding among healthcare professionals can have due to the resulting misdiagnosis and the pressure it can place on autistic adults. This is in direct opposition to the values of the UK healthcare system which values ‘improving lives’ (Department of Education, & Department of Health and Social Care, 2021) and to ‘do no harm’ (Welsh Government, 2016). Therefore, it is recommended that the provision of training co-produced with autistic adults to all healthcare professionals across the board who will come into contact with autistic patients is prioritised. The provision of different methods of communication and alternative quiet waiting areas should also be considered.

### Limitations

There is still limited research completed on UK-specific experiences, and the studies included covered a range of different settings with a number focused on the diagnostic pathway and mental health services, but with several standing alone as representatives in their field for optometry, dentistry, perinatal and eating disorder services. The majority of the existing studies were focused on the mental health setting, and so results are less transferable to the wider healthcare system. While the themes across the papers were common, suggesting the same issues tend to arise regardless of the setting, further research into the experiences of autistic adults in physical healthcare settings is required.

The studies included in this meta-ethnography cover the experiences of autistic adults living across the United Kingdom, with some specifically focused on parts of England or Wales. It is important to note that NHS England and NHS Wales have different ways of working and therefore although this analysis offers the first focused look at experiences specific to the UK healthcare system, results may not necessarily be generalisable from one health board to another. This meta-synthesis also cannot offer recommendations on the healthcare experiences of autistic children in the United Kingdom, or of those autistic adults’ with co-occurring learning disabilities, and this is an area which may benefit from further research.

A further limitation of this article is that while one of the lead authors is an autistic adult, it would have been beneficial to have more involvement of another autistic adult in the design and interpretation of findings.

## Conclusion

This meta-ethnography highlights difficulties experienced by autistic adults without a co-occurring learning disability in the United Kingdom when accessing healthcare services. This review offers new insights into the wide-reaching damaging impact that a lack of autism knowledge and understanding among healthcare professionals can have on well-being. Such damage occurs in the context of misdiagnosis, inadequate or inappropriate treatment, overwhelming environments and inaccessible systems. This can be addressed through more comprehensive and widely delivered training co-produced with autistic service users, alongside bespoke and person-centred adaptations.
